# Multiplex testing for Factor II and Factor V mutations in thrombophilia: technical verification and clinical validation of the cobas® Factor II and Factor V test

**DOI:** 10.1007/s11239-018-1745-8

**Published:** 2018-10-03

**Authors:** John W. Longshore, Kelli DeMartin, Karen Yu, Partha Das, Guili Zhang, Taraneh Tamaddon Rehage, Deepa Jethwaney, Sylwia Karwowska

**Affiliations:** 1 Carolinas Pathology Group and Carolinas HealthCare System, Charlotte, NC USA; 2 Roche Molecular Systems Inc, 4300 Hacienda Drive, Pleasanton, CA 94588 USA

**Keywords:** Thrombophilia, **cobas®** Factor II and Factor V test, Factor II G20210A, Factor V Leiden

## Abstract

Laboratory testing for thrombophilia is complicated but essential for diagnosis. In 2017, the **cobas**® Factor II and Factor V Test (cobas F2F5 test) was launched for use with the **cobas z** 480 analyzer. This qualitative polymerase chain reaction test enables multiplex Factor II and Factor V testing with flexible reporting and workflow efficiency. Here, we report the results from studies investigating the performance of the cobas F2F5 test. Technical performance verification, clinical validation, external laboratory performance, and workflow comparison studies were performed. Fresh and frozen whole-blood and genomic DNA (gDNA) samples were tested, and several manual and automated DNA isolation methods were used. Bidirectional Sanger sequencing was used to verify genotypes identified by the cobas F2F5 test. One hundred percent agreement between the cobas F2F5 test and Sanger sequencing was observed for all genotypes. An external laboratory using remnant clinical samples also yielded 100% agreement between cobas F2F5 test results and their routine testing method. The cobas F2F5 test reduced the total sample processing time compared with the LightCycler® 1.2 platform (98.6 vs 420.2 min; 96 samples). Hemoglobin, extraction buffer, and ethanol contamination of the gDNA sample can lead to invalid results. The cobas F2F5 test has a high degree of accuracy for identification of Factor II and Factor V genotypes. This multiplex testing with short sample processing time can reduce handling errors and increase efficiency. Both manual and automated DNA isolation methods can be used with the cobas F2F5 test.

**Electronic supplementary material:**

The online version of this article (10.1007/s11239-018-1745-8) contains supplementary material, which is available to authorized users.

## Key messages

- Roche Molecular Systems recently launched a new real-time polymerase chain reaction test, the **cobas**® Factor II and Factor V Test, to detect common mutations associated with thrombophilia [13]
- The cobas F2F5 test allows for multiplex testing with flexible reporting and a user-selected sample DNA extraction method
- Technical performance and clinical validation studies found 100% agreement between genotypes reported by the cobas F2F5 test and reference Sanger sequencing
- Compared with the LightCycler® 1.2 platform–based testing method, the cobas F2F5 test also reduces the total processing time and number of steps required in amplification and detection
- These results show that the new cobas F2F5 test is both time saving and cost-efficient and provides a high level of accuracy in Factor II and Factor V genotype identification

## Introduction

Thrombophilia, characterized by a predisposition to the development of thrombi in veins, arteries, or both, is the result of either inherited or acquired defects (or an interaction between the 2) in the coagulation system [1, 2]. The most commonly associated genetic mutations for inherited thrombophilia are gain-of-function mutations in the genes for Factor V and Factor II (prothrombin). The American College of Medical Genetics and recent guidelines from other professional societies recommend genetic testing only in certain circumstances for patients with suspected hereditary venous thrombosis [3, 4]. Patients who test positive for Factor V Leiden should be considered for Factor II G20210A testing, particularly pregnant women with a previous history of venous thromboembolism and recurrent pregnancy loss [3–5].

Several different laboratory testing methods are currently available for Factor II and Factor V mutation detection [6]. Based on internal data from market assessments and sales, we estimate that approximately 2.2 million Factor II and Factor V genotyping tests are performed annually worldwide (excluding Japan) [7]. The laboratory costs of thrombophilia testing are estimated to exceed $650 million (USD) annually [8]. Therefore, there is an urgent need to optimize screening and diagnosis algorithms, make testing more cost-effective for laboratories, and reduce the frequency of inappropriate testing [9, 10].

**Fig. 1:**
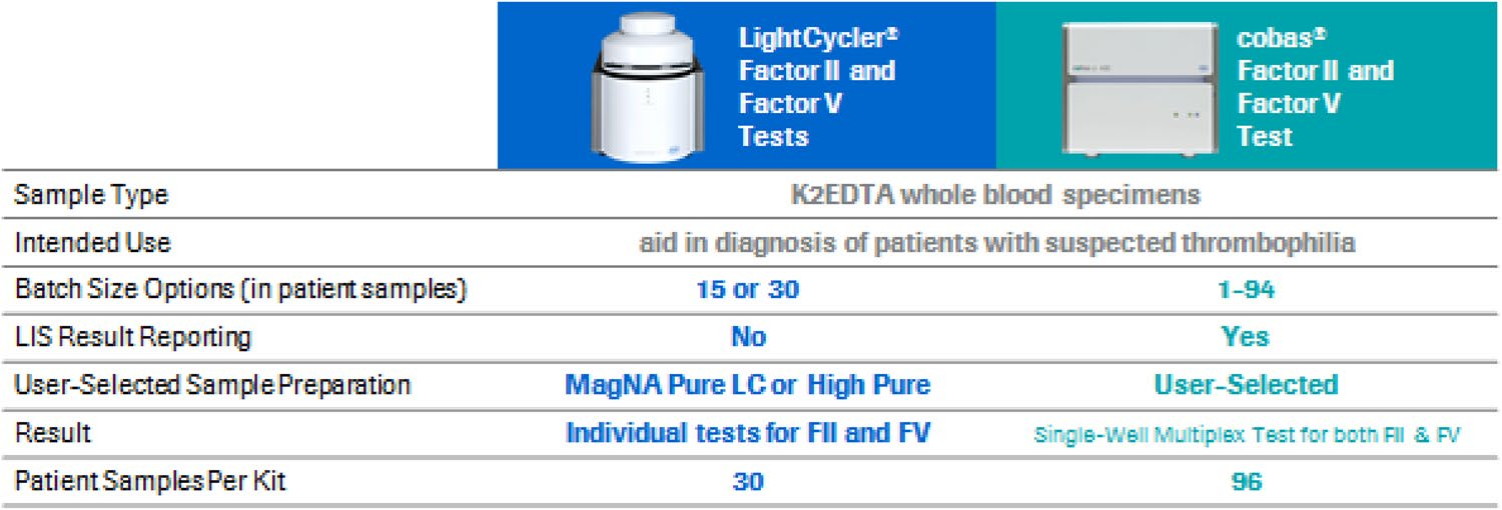
Comparison of the 2 Roche Molecular Systems qualitative polymerase chain reaction (qPCR) methods for Factor II and Factor V detection using the LightCycler and **cobas z** 480 instruments. *FII* Factor II, *FV* Factor V, *K*_*2*_*EDTA* dipotassium ethylenediaminetetraacetic acid, *LIS* laboratory information system

In 2003, Roche Molecular Diagnostics launched 2 qualitative real-time polymerase chain reaction (PCR)-based diagnostic tests for use with the LightCycler® 1.2 Instrument—the Factor II G20210A Kit and the Factor V Leiden Kit—each allowing for detection and genotyping of a single point mutation from whole-blood genomic DNA (gDNA) [11, 12]. In 2017, keeping with evolving laboratory practices and technological advances, the **cobas®** Factor II and Factor V Test (cobas F2F5 test) was launched for use with the **cobas z** 480 analyzer. This test not only allows for multiplex testing but also flexible reporting—either both mutations together or individually—depending on testing requirements. The testing algorithm was optimized to distinguish between wild-type and mutant alleles and designed to prevent the occurrence of incorrect results. For greater flexibility prior to amplification and detection, DNA extraction from whole blood can be performed offline using a user-selected manual or automated method. Multiplex testing also improves cost-efficiency by reducing the testing time and minimizing the risk of error. Figure 1 summarizes the differences between the earlier LightCycler and the new **cobas z** 480 platforms.

Here, we report the results from technical performance verification, clinical validation, and external laboratory performance studies for the cobas F2F5 test.

## Methods

**Fig. 2:**
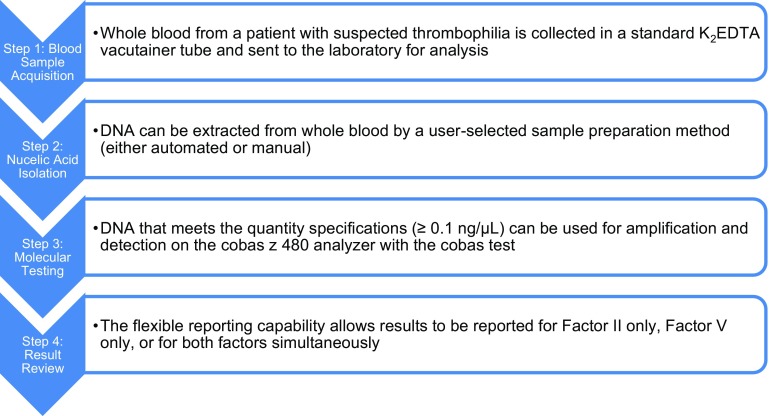
Overall workflow for Factor II and Factor V identification using the **cobas z** 480 platform. *K*_*2*_*EDTA* dipotassium ethylenediaminetetraacetic acid

The overall workflow for Factor II and Factor V testing using the cobas F2F5 test platform is summarized in Fig. 2.

### Technical performance verification

As part of the development of the cobas F2F5 test, studies were designed to investigate the performance characteristics of the test, such as the limit of detection, analytical specificity, stability (including samples and reagents), and substances that may interfere with the performance of the test. Further details about the studies are provided in Supplementary Table 1.

### Clinical validation studies

#### Method comparison

Frozen whole-blood and DNA specimens (n = 300) were obtained from laboratories that performed Factor II and/or Factor V testing per routine medical care. Genotypes tested included Factor II or Factor V homozygous mutations, single or compound heterozygous mutations, and wild-type samples. Factor II homozygous mutant samples (n = 16) were available as gDNA only. Manual gDNA isolation using the High Pure (HP) PCR Template Preparation Kit (Roche Molecular Systems Inc, Pleasanton, CA) and cobas F2F5 testing were carried out at an external site. Bidirectional Sanger DNA sequencing was carried out at a commercial laboratory.

#### Clinical reproducibility

Nine panel members were tested at three sites, with each site using a different commercially available manual DNA extraction kit: HP kit (Roche Molecular Systems Inc, Pleasanton, CA), ReliaPrep™ Blood gDNA Miniprep System (Promega, Madison, WI), and QIAamp® DSP DNA Blood Mini Kit (Qiagen, Venlo, the Netherlands). The nine panel members tested consisted of four dipotassium ethylenediaminetetraacetic acid (K_2_EDTA) whole-blood samples, three contrived whole-blood samples, and two gDNA samples (diluted to 0.2 ng/µL). Each testing site had two operators and one instrument. Each operator performed 1 run per day over 5 non-consecutive days. A total of 18 cobas F2F5 tests were carried out for each panel member (in duplicate) for each reagent lot.

### External laboratory performance testing

Performance of the cobas F2F5 test in combination with automated DNA extraction methods was assessed using frozen and fresh clinical samples. Samples were de-identified prior to processing and testing. Banked (frozen) whole-blood specimens (n = 200)—a subpopulation of samples used for the method comparison clinical validation study (using manual DNA isolation; outlined above)—were provided by Roche Molecular Systems. gDNA was isolated using both the MagNA Pure 24 (MP 24) and MagNA Pure 96 (MP 96) automated DNA isolation methods. Overall percentage agreement (OPA) was assessed against archived Sanger sequencing results and between DNA isolation methods. Remnant clinical samples (fresh, never frozen; n = 200) from routine testing were provided by an external study site and DNA was isolated using the MP 96 isolation method. The cobas F2F5 test results were compared with results from routine Factor II and Factor V testing methods used at the external site (Roche Factor II prothrombin G20210A kit and Roche Factor V Leiden Kit coupled to the LightCycler® 1.2 instrument).

### Workflow comparison

**Fig. 3:**
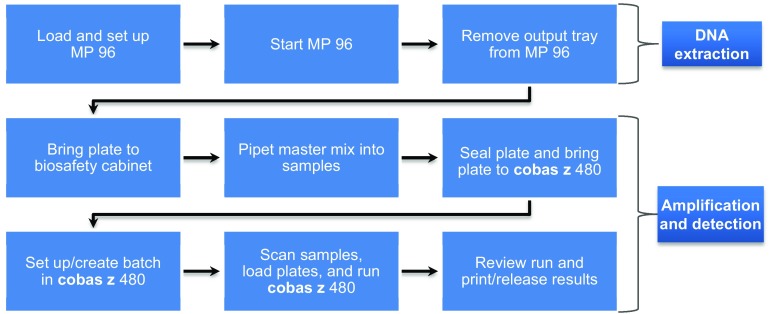
Overview of the workflow using the MP 96 and **cobas z** 480 platform for the **cobas**® Factor II and Factor V Test. MP 96, MagNA Pure System

The workflow for cobas Factor II and Factor V testing using the **cobas z** 480 platform was compared with the LightCycler® 1.2 platform. The hands-on and total processing times in addition to the number of steps from specimen receipt to release of results were observed and documented for different batch sizes. DNA isolation was carried out using the MP 96 instrument prior to testing with both platforms. Figure 3 shows representative steps in the workflow comparison study using the example of the MP 96 instrument paired with the **cobas z** 480 for the new cobas F2F5 test (MP 96 paired with LightCycler steps not shown).

### Study ethics

All studies were conducted in compliance with their protocols. Clinical studies were conducted with the International Conference on Harmonisation Good Clinical Practice Guidelines and regulations of the US Food and Drug Administration (FDA). Where appropriate, study protocols were submitted to an institutional review board in accordance with FDA and local regulatory requirements prior to the start of the study.

## Results

### Technical performance verification studies

All studies met predefined (prior to test development) acceptance criteria. The aims and key results from each technical performance study are outlined in Table 1.

**Table 1:** Summary of results from technical performance verification studies for the cobas F2F5 test

Study type	Name	Aim and key findings
DNA isolation	DNA extraction method study	*Aim* To determine the performance of the cobas F2F5 test on gDNA isolated from K_2_EDTA whole blood using 3 different commercially available kits *Result* For each DNA isolation method, the cobas F2F5 test yielded 100% correct results when compared with bidirectional Sanger sequencing One of the gDNA samples isolated was rust colored and yielded invalid results (see Discussion)
Specificity	Target sequence exclusivity study	*Aim* To determine the exclusivity of the cobas F2F5 test for the Factor II prothrombin and Factor V Leiden mutations and wild-type alleles *Result* BLAST searching of the EMBL nucleotide sequence database and human genome did not identify any sequences with the potential to cross-react or interfere with the cobas F2F5 test
Interference and contamination	Potentially interfering substances study	*Aim* To determine the impact of potentially interfering substances present in whole blood or gDNA on the performance of the cobas F2F5 test *Result* Correct genotype results were obtained for 6 whole-blood specimens that were tested with a panel of interfering substances Ethanol (5% v/v) and extraction buffer (2.5% v/v) added to gDNA interfered with the cobas F2F5 test and caused invalid results (see Discussion) Current standard-of-care blood-thinner drugs (eg, heparin, warfarin, rivaroxaban, dabigatran etexilate) did not interfere with the test results
	Potentially interfering mutations study	*Aim* To determine the effect of known SNPs close to the Factor II 20210 or Factor V 1691 locus on the detection of the Factor II 21210G > A and Factor V 1691G > A mutations using the cobas F2F5 test *Result* None of the SNP plasmids caused false-positive results. All but one of the SNPs were detected as wild-type. The Factor V 1689G > A mutation was not detected by the cobas F2F5 test when present on both alleles (see Discussion)
	Cross-contamination study	*Aim* To determine the frequency of cross-contamination during manual gDNA isolation and execution of the cobas F2F5 test *Result* The cross-contamination rate was 0%
Stability	Whole-blood stability study	*Aim* To determine the stability of K_2_EDTA whole-blood specimens To compare the performance of the cobas F2F5 test on gDNA isolated from freshly drawn whole-blood samples vs whole blood that was frozen and thawed up to three times *Result* Correct results were obtained for all samples, storage conditions, and time intervals tested, including up to 3 freeze/thaw cycles at − 20 °C and ≤ − 70 °C for whole blood K_2_EDTA whole-blood samples may be stored at 2 °C–8 °C for up to 7 days, 25 °C–30 °C for up to 3 days, or frozen at − 20°C and ≤ −70 °C for up to 8 months
	gDNA stability study	*Aim* To determine the stability of gDNA isolated from K_2_EDTA whole-blood specimens To compare the performance of the cobas F2F5 test on gDNA (isolated using three commercially available sample preparation methods) from freshly drawn whole-blood samples vs frozen whole blood that was thawed up to three times *Result* Correct results were obtained for all samples, storage conditions, sample preparation methods, and time intervals tested, including up to 3 freeze/thaw cycles at − 20 °C and ≤ −70 °C for gDNA gDNA isolated from K_2_EDTA whole-blood samples may be stored at 2 °C–8 °C for up to 7 days, or frozen at − 20 °C and ≤ −70 °C for up to 12 months
	Open reagent stability study	*Aim* To determine the stability of opened reagents (including controls) of the cobas F2F5 test after they have been used once *Result* Kit reagents are stable at 2 °C–8 °C for at least 90 days for up to two uses (only two uses were tested)
	Activated MMX stability study	*Aim* To determine the stability of activated MMX at room temperature (MMX is activated when combined with the cofactor) *Result* Activated MMX stability acceptance criteria were met at all tested time intervals. Activated MMX may be stored at room temperature for at least 4 h
	Activated MMX plus extracted sample stability study	*Aim* To determine the stability of activated MMX combined with processed specimens (gDNA isolated from whole blood) at room temperature (up to 30 °C) *Result* All tests of all samples yielded correct results when compared with bidirectional Sanger sequencing. Activated MMX with isolated DNA or run controls is stable for at least 2 h at room temperature
Analytical sensitivity and DNA concentration	Lower limit of analytical sensitivity	*Aim* To determine the limit of detection and minimum input gDNA concentration for the cobas F2F5 test *Result* The lowest input gDNA concentration with at least ≥ 95% correct genotype results was 0.01 ng/µL The recommended lowest gDNA input concentration is 0.1 ng/µL (see Discussion)
	Upper limit of analytical sensitivity	*Aim* To determine the maximum concentration of gDNA input for the cobas F2F5 test *Result* Correct results were observed with gDNA input as high as 300 ng/µL. The recommended highest gDNA input concentration is 150 ng/µL (see Discussion)
	Validation of DNA concentration from whole-blood specimens	*Aim* To validate the recommended quantity and quality specifications for gDNA from > 300 K_2_EDTA whole blood as determined by UV spectroscopy after isolation using various commercially available DNA isolation methods *Result* cobas F2F5 test results agreed with Sanger sequencing for all samples that met the following specifications 95% of gDNA samples had a DNA concentration ≥ 2 ng/µL and 99% of gDNA samples had a DNA concentration ≥ 0.2 ng/µL

*BLAST* basic local alignment search tool, *cobas F2F5 test***cobas**® Factor II and Factor V test, *EMBL* European Molecular Biology Laboratory, *gDNA* genomic DNA, *K*_*2*_*EDTA* dipotassium ethylenediaminetetraacetic acid, *MMX* master mix, *SNP* single-nucleotide polymorphism

### Clinical validation studies

#### Method comparison

All runs and sample results were valid (no repeat tests were needed). A genotype result was classified as correct if the same genotype was detected by both the cobas F2F5 test and the reference method, bidirectional Sanger sequencing.

For Factor II testing (n = 300), the OPA with bidirectional Sanger sequencing was 100%, with a 2-sided 95% lower confidence boundary (LCB; exact method) of 98.78%. The negative percentage agreement (NPA; for wild-type samples) was 100% (95% LCB, 97.55%; n = 149). The positive percentage agreement (PPA; for Factor II mutation-positive samples) was also 100% (95% LCB, 97.59%; n = 151). Of the 151 positive samples, the percentage agreement for both heterozygous and homozygous mutant samples was 100% each (95% LCB, 97.20%; n = 130 and 95% LCB, 83.89%; n = 21, respectively).

Similarly, for Factor V testing, the OPA was 100% (95% LCB, 98.78%; n = 300), NPA was 100% (95% LCB, 97.52%; n = 147), and PPA was 100% (95% LCB, 97.62%; n = 153). For the positive samples, the percentage agreement for both heterozygous and homozygous mutant samples was 100% each (95% LCB, 97.20%; n = 130 and 95% LCB, 85.18%; n = 23, respectively).

#### Clinical reproducibility

Four hundred twenty whole-blood samples (240 clinical and 180 contrived) and 120 gDNA samples were tested. A total of 540 tests obtained from 30 valid runs yielded 539 valid results. One invalid result was due to a lack of agreement in the data parameter checks for software result interpretation, designed to prevent inaccurate result reporting. The invalid rate for whole-blood samples in the study was 0.24% (1 of 420).

The cobas F2F5 test demonstrated 100% correct call rates for each Factor II and Factor V genotype tested (Table 2).

**Table 2:** Summary of results from the reproducibility study by site, lot, sample preparation method, and genotype for Factor II and Factor V testing using the **cobas**® Factor II and Factor V Test

Site/lot/sample preparation method	Genotype panel	Total samples tested	Correct calls	Percentage agreement (95% LCB^a^)
Reproducibility study for Factor II				
Site 1/Lot 1/Roche High Pure PCR Template Preparation Kit	WT	100	100	100.0 (97.05)
	HET	60	60	100.0 (95.13)
	MUT	20	20	100.0 (86.09)
Site 2/Lot 2/Promega ReliaPrep™ Blood gDNA Miniprep System	WT	100	100	100.0 (97.05)
	HET	60	59^b^	100.0 (95.05)
	MUT	20	20	100.0 (86.09)
Site 3/Lot 3/Qiagen QIAamp® DSP DNA Blood Mini Kit	WT	100	100	100.0 (97.05)
	HET	60	60	100.0 (95.13)
	MUT	20	20	100.0 (86.09)
Reproducibility Study for Factor V				
Site 1/lot 1/Roche HIAGH pure PCR template preparation kit	WT	100	100	100.0 (97.05)
	HET	60	60	100.0 (95.13)
	MUT	20	20	100.0 (86.09)
Site 2/Lot 2/Promega ReliaPrep Blood gDNA Miniprep System	WT	100	100	100.0 (97.05)
	HET	60	59^b^	100.0 (95.05)
	MUT	20	20	100.0 (86.09)
Site 3/Lot 3/Qiagen QIAamp DSP DNA Blood Mini Kit	WT	100	100	100.0 (97.05)
	HET	60	60	100.0 (95.13)
	MUT	20	20	100.0 (86.09)

*gDNA* genomic DNA, *HET* heterozygous, *LCB* lower confidence bound, *MUT* homozygous mutant, *WT* wild-type

^a^Overall percentage agreement with 95% lower confidence bound of 98.17% (2-sided 95% LCB was calculated using the exact method)

^b^Invalid result that was not retested

### External laboratory performance testing

For banked (frozen) specimens, the cobas F2F5 test results agreed with the genotypes determined by Sanger sequencing for 100% of all genotypes tested regardless of the DNA isolation method used, manual or automated (n = 200; 95% LCB, 98.2%). For Factor II genotypes, the percentage agreement was 100% each for wild-type (n = 103), heterozygous mutant (n = 94), and homozygous mutant (n = 3) samples. For Factor V genotypes, the percentage agreement was also 100% each for wild-type (n = 87), heterozygous mutant (n = 96), and homozygous mutant (n = 17) samples.

Results from remnant whole-blood specimens tested with the MP 96 platform showed that the cobas F2F5 test generated 100% correct calls and 100% OPA compared with the LightCycler method for all Factor II and Factor V genotypes tested (95% LCB, 98.17%; n = 200). For Factor II genotypes, the percentage agreement was 100% for both wild-type (n = 196) and heterozygous mutant (n = 4) samples (no homozygous mutant samples were tested). Similarly, for Factor V genotypes, the percentage agreement was 100% each for wild-type (n = 178), heterozygous mutant (n = 21), and homozygous mutant (n = 1) samples.

### Workflow overview study

Results for the hands-on and total processing times in the pre-analytic (gDNA isolation) phase are provided in Supplementary Table 2.

Compared with the LightCycler® 1.2–based method, the cobas F2F5 test required less hands-on time (141.2 vs 8.6 min), less total processing time (420.2 vs 98.6 min), and fewer steps for amplification and detection (1091 vs 303; n = 96 samples). The processing times for 24 and 48 samples are provided in Supplementary Table 2.

## Discussion

In this paper, we report results from several studies evaluating the performance characteristics of the new cobas F2F5 test for thrombophilia. In all, these studies show that the cobas F2F5 test is robust and has a very high degree of accuracy in determining Factor II and Factor V genotypes from frozen and fresh whole-blood and gDNA samples.

The minimum recommended gDNA input for the cobas F2F5 test is 0.1 ng/μL, which is close to that observed for > 99% of gDNA samples used in our testing (≥ 0.2 ng/μL). However, correct results were generated at a concentration that is 10 times lower than recommended (Table 1). The maximum recommended gDNA concentration is 150 ng/μL, which is a conservative recommendation since correct results were generated using a concentration up to 300 ng/μL (Table 1). No incorrect genotypes were reported in these studies.

One gDNA plasma sample with a rust color, most likely due to hemoglobin (a polymerase chain reaction inhibitor), yielded an invalid result in the DNA extraction method study (Table 1). It is recommended, therefore, that the appearance of gDNA isolated from whole blood should be clear and colorless; samples that contain a red-colored tint may yield invalid or incorrect results. Extraction buffers and ethanol in commercial DNA isolation kits can also cause invalid results (Table 1). These buffers typically contain chaotropic salts (eg, guanidine hydrochloride), which are potent denaturants and inhibit DNA polymerase. However, any residual extraction buffer is typically removed during the wash steps between lysis and elution in commercial DNA isolation kits. Ethanol is a common ingredient in the final wash buffer, and it is recommended that both the lysis and wash buffers are completely removed from the sample before running the cobas F2F5 test.

Eight known single-nucleotide polymorphisms close to the Factor II or Factor V locus were tested with the cobas F2F5 test and no false positives were detected. In the rare event that a Factor V 1689 G > A mutation is present on both Factor V alleles, the cobas F2F5 test will yield an invalid result. All the aforementioned considerations are included in the **cobas®** Factor II and Factor V Test kit *Instructions for Use*.

In these studies, a variety of commercially available manual and automated DNA isolation methods were used, and the cobas F2F5 test identified Factor II and Factor V genotypes with a high degree of accuracy. The reproducibility study found 100% agreement between the cobas F2F5 test and Sanger sequencing for all genotypes and manual DNA isolation methods tested. Similarly, external laboratory performance testing found 100% agreement for all frozen and whole-blood specimens isolated using both manual and automated DNA extraction methods. These results highlight the “open front-end” capability of the cobas F2F5 test, which allows the user to select any gDNA isolation method, provided it meets the recommended minimum gDNA quantity [13].

Compared with the preceding LightCycler® 1.2 platform, the cobas F2F5 test and **cobas z** 480 platform reduced the hands-on and total processing times for 96 samples by 16- and fourfold, respectively. The total number of steps was also reduced by approximately fourfold, decreasing the likelihood of user processing errors.

Factor II and Factor V mutation testing is expensive, time consuming [7, 10], and associated with errors that can lead to misdiagnosis or overtreatment [14]. The Italian Committee for the Standardization of Laboratory Tests reported a 4.9–17.0% error rate for Factor V Leiden and 4.9–19.5% error rate for Factor II G20210 identification [15]. The Royal College of Pathologists of Australasia reported a 98.63% success rate for Factor V and Factor II mutation identification but found large variations between laboratories: 51% made ≥ 1 error and 3 of 39 laboratories were responsible for nearly half the reported errors [16]. Results of genetic analyses can impact clinical decision making and patient levels of concern, thus testing laboratories should adhere to a high level of internal quality control and participate in external quality assurance programs [16].

In conclusion, the new cobas F2F5 test can improve the efficacy of thrombophilia testing through its established accuracy in genotype identification. In addition, the multiplex method and short processing time can reduce the cost and time associated with Factor II and Factor V testing. Lastly, the flexible reporting and “open front-end” design can enable easy integration into established testing procedures in laboratories.

## Electronic supplementary material

Below is the link to the electronic supplementary material.

Supplementary material 1 (DOCX 49 KB)
