# Supplementary material for: Multiplex testing for Factor II and Factor V mutations in thrombophilia: technical verification and clinical validation of the cobas® Factor II and Factor V test
Source: J Thromb Thrombolysis. 2018 Oct 3;47(1):87–95. doi: 10.1007/s11239-018-1745-8 (PMC6336749; doi:10.1007/s11239-018-1745-8)
Supplement: Supplementary file 1 — Supplementary material 1 (DOCX 49 KB) [file 11239_2018_1745_MOESM1_ESM.docx]

**Supplementary Materials**

**Multiplex testing for Factor II and Factor V mutations in thrombophilia: technical verification and clinical validation of the cobas^®^ Factor II and Factor V Test**

*J Thromb Thrombolysis*

John W. Longshore,^1^ Kelli DeMartin,^2^ Karen Yu,^2^ Partha Das,^2^ Guili Zhang,^2^ Taraneh Tamaddon Rehage,^2^ Deepa Jethwaney,^2^ Sylwia Karwowska ^2^

^1^ Carolinas Pathology Group and Carolinas HealthCare System, Charlotte, North Carolina, USA

^2^ Roche Molecular Systems Inc, Pleasanton, California, USA

**Corresponding author:**

Sylwia Karwowska, Roche Molecular Systems Inc, 4300 Hacienda Drive, Pleasanton, CA 94588, USA, sylwia.karwowska@roche.com

**Supplementary Table 1** Summary of methods for each of the technical performance verification studies performed as part of the development of the cobas F2F5 test

| TPV Study Name | Method |
| --- | --- |
| DNA extraction method | - gDNA from 15 whole-blood and contrived whole-blood specimens was isolated using 3 commercially available kits: Roche HP kit, Qiagen QIAamp^®^ DSP DNA Blood Mini Kit and Promega ReliaPrep^TM^ Blood gDNA Miniprep System - Genotypes included wild-type, heterozygous, and homozygous genotypes for Factor II and Factor V - DNA was isolated from each blood specimen using 1 method per day by 2 operators. Each isolated gDNA sample was tested 3 times by the cobas F2F5 test for a total of 18 results from 6 DNA isolations for each specimen - Factor II and Factor V genotype results were compared with the expected genotypes determined by bidirectional Sanger sequencing |
| Target sequence exclusivity | - Nucleic acid sequence homology BLAST searches were performed on the EMBL nucleotide sequence database and human genome sequence to identify sequences in the human genome (or microorganisms present in human blood) with homology or partial homology to the primers and probes of the cobas F2F5 test |
| Potentially interfering substances | - Six K_2_EDTA whole-blood were tested using the cobas F2F5 test in the presence and absence of potentially interfering substances - The following were added to whole-blood specimens: bilirubin (conjugated and unconjugated), hemoglobin, cholesterol, triglycerides, heparin, warfarin, rivaroxaban, dabigatran etexilate, and K_2_EDTA. Extraction buffer and ethanol were added to gDNA eluates |
| Potentially interfering mutations | - Eight known SNPs (20207A>C, 20209C>T, 20218A>G, 20221C>T, 1689G>A, 1690C>T, 1692A>C, and 1696A>G) were tested - Linearized plasmid DNA for each SNP was tested under 4 different conditions (alone or in combination with Factor II and Factor V wild-type control plasmids and gDNA from wild-type whole blood) to simulate different Factor II and Factor V genotype combinations |
| Cross-contamination | - Five runs of DNA isolation and the cobas F2F5 test with 12 replicates each of a unique K_2_EDTA compound heterozygous whole-blood sample and a negative control sample were tested in each run - During gDNA isolation, whole-blood and negative control samples were arranged in alternating positions on the tube racks and in the centrifuge rotor - gDNA from whole-blood specimens and negative controls were added to the cobas amplification/detection plates in a checkerboard pattern |
| Whole-blood stability | - K_2_EDTA whole-blood specimens were stored at 2°C-8°C, 25°C, 32°C, −20°C, and ≤ −70°C. Frozen samples were subjected to 1, 2, or 3 freeze/thaw cycles - At different testing intervals, gDNA was isolated and immediately tested. Genotypes were confirmed by bidirectional Sanger sequencing |
| Open reagent stability | - The stability of opened vials was determined by removing half the volume from multiple vials of the kit’s reagents and controls, recapping and storing the vials at 2°C-8°C, and testing them after approximately 31, 61, and 91 days |
| Activated MMX stability | - Activated MMX was prepared and tested after storage at 32°C for 0, 1, 2, 2.5, 4, and 4.5 hours - Functional testing was performed at each time interval using 4 K_2_EDTA whole-blood specimens |
| Activated MMX plus extracted sample stability | - gDNA was isolated in triplicate from 4 K_2_EDTA whole-blood specimens - Five runs were prepared by adding activated MMX to the wells of the cobas amplification/detection plate. gDNA and positive and negative controls were then added. One run was performed immediately (T0) and 4 additional runs were performed after incubation of the plates at 32°C for 1, 1.5, 2, and 2.5 hours |
| Lower limit of analytical sensitivity | - Serial dilutions were prepared from gDNA isolated from 3 K_2_EDTA whole-blood specimens (Factor II heterozygous, Factor V heterozygous, and Factor V homozygous mutant) and 1 cell line (Factor II homozygous mutant) - Each sample was tested at 10 concentrations: undiluted (6-38 ng/μL) and 9 serial dilutions from 1.0-0.0001 ng/μL |
| Upper limit of analytical sensitivity | - High-concentration gDNA samples were prepared using 2 methods - Method 1: “High-input” and “standard” gDNA were extracted using 3 commercial isolation kits. “High-input” gDNA was isolated by using double the standard input volume of whole blood and 30% of the standard elution buffer volume. “Standard” gDNA was isolated using the standard volumes of whole blood and elution buffer. - Method 2: Contrived samples were prepared by isolating gDNA from 4 K_2_EDTA whole-blood samples using 3 commercially available DNA extraction methods and adding concentrated DNA from cell lines of the same genotype. The final DNA concentrations were approximately 300, 150, and 75 ng/μL |
| Validation of DNA concentration from whole-blood specimens | - A total of 606 gDNA samples were isolated from 321 unique K_2_EDTA whole-blood specimens using 3 different DNA isolation methods (Roche HP kit, QIAgen QIAamp^®^ DSP DNA Blood Mini Kit, and Promega ReliaPrep^TM^ Blood gDNA Miniprep System). Additionally, 16 samples were obtained as isolated gDNA from unknown isolation methods - The concentration of gDNA samples was determined by measuring absorbance at 260 nm after results of the cobas F2F5 test were obtained. Genotypes identified by the cobas F2F5 test were compared against results from bidirectional Sanger sequencing |

BLAST, Basic Local Alignment Search Tool; cobas F2F5 test, **cobas**^®^ Factor II and Factor V Test; EMBL, European Molecular Biology Laboratory; gDNA, genomic DNA; HP kit, High Pure PCR Template Preparation Kit; K_2_EDTA, dipotassium ethylenediaminetetraacetic acid; MMX, master mix; SNP, single-nucleotide polymorphism; TPV, technical performance verification

**Supplementary Table 2** Results from the workflow comparison study comparing the processing time for the LightCycler^®^ 1.2 platform–based Factor II and Factor V identification vs the cobas^®^ Factor II and Factor V Test. The hands-on and total processing times and total number of steps are reported for 3 different sample sizes

| **Workflow** | **Platform** | **Processing Time** | | | | | | | | |
| --- | --- | --- | --- | --- | --- | --- | --- | --- | --- | --- |
|  |  | **24 Samples** | | | **48 Samples** | | | **96 Samples** | | |
|  |  | **Hands-On Time, min** | **Total Processing Time, min** | **Total Steps** | **Hands-On Time, min** | **Total Processing Time, min** | **Total Steps** | **Hands-On Time, min** | **Total Processing Time, min** | **Total Steps** |
| Pre-analytics | MP 96 | 22.9 | 82.9 | 106 | 43.3 | 103.3 | 202 | 84.1 | 144.1 | 394 |
| Amplification and detection | cobas z 480 | 3.7 | 93.7 | 87 | 5.3 | 95.3 | 159 | 8.6 | 98.6 | 303 |
|  | LightCycler 1.2^®^ | 42.2 | 134.7 | 299 | 75.2 | 260.2 | 563 | 141.2 | 420.2 | 1091 |

MP 96, MagNA Pure 96
